# Cellular Strategies for Frequency-Dependent Computation of Interaural Time Difference

**DOI:** 10.3389/fnsyn.2022.891740

**Published:** 2022-05-06

**Authors:** Rei Yamada, Hiroshi Kuba

**Affiliations:** Department of Cell Physiology, Graduate School of Medicine, Nagoya University, Nagoya, Japan

**Keywords:** interaural time difference, coincidence detection, synapse, dendrite, ion channel

## Abstract

Binaural coincidence detection is the initial step in encoding interaural time differences (ITDs) for sound-source localization. In birds, neurons in the nucleus laminaris (NL) play a central role in this process. These neurons receive excitatory synaptic inputs on dendrites from both sides of the cochlear nucleus and compare their coincidences at the soma. The NL is tonotopically organized, and individual neurons receive a pattern of synaptic inputs that are specific to their tuning frequency. NL neurons differ in their dendritic morphology along the tonotopic axis; their length increases with lower tuning frequency. In addition, our series of studies have revealed several frequency-dependent refinements in the morphological and biophysical characteristics of NL neurons, such as the amount and subcellular distribution of ion channels and excitatory and inhibitory synapses, which enable the neurons to process the frequency-specific pattern of inputs appropriately and encode ITDs at each frequency band. In this review, we will summarize these refinements of NL neurons and their implications for the ITD coding. We will also discuss the similarities and differences between avian and mammalian coincidence detectors.

## Introduction

A neuron is a computational unit in the brain. It receives and integrates synaptic inputs at dendrites and the soma, and generates action potentials in the axon, thereby communicating with other neurons. These neuronal compartments show huge diversity among neuronal types and brain regions. They include variations in length, thickness, branching patterns of dendrites, distributions of ion channels as well as excitatory and inhibitory synapses, all of which finely tune the computation of individual neurons and play a key role in determining the function of neural circuits and the brain (Shepherd, 2004).

In the auditory system, sound information is processed after the sound is divided into each frequency component in the cochlea and converted into a sequence of action potentials in the auditory nerve, where the frequency is represented as the selective activity in the fibers tuned to a specific frequency (characteristic frequency, CF), phase as the timing of action potentials phase-locked to the sound wave, and intensity as the number of action potentials in the fibers. Accordingly, the processing of sound in central auditory circuits is operated for each frequency separately by neurons with different CFs, which are arranged topographically in circuits (tonotopy). These results imply that the temporal profiles of synaptic input to auditory neurons differ substantially among tonotopic regions. To ensure precise signal processing across intensities in each tonotopic region, auditory neurons show multiple frequency-specific refinements in the morphological and biophysical characteristics of dendrites and other neuronal compartments. Such refinements have been studied extensively in brainstem circuits that are involved in the calculation of the interaural time difference (ITD) for sound localization in birds, primarily chickens that have excellent sound localization ability (Krumm et al., 2022).

## Frequency-Dependent ITD Coding in the Nucleus Laminaris

**Figure 1:**
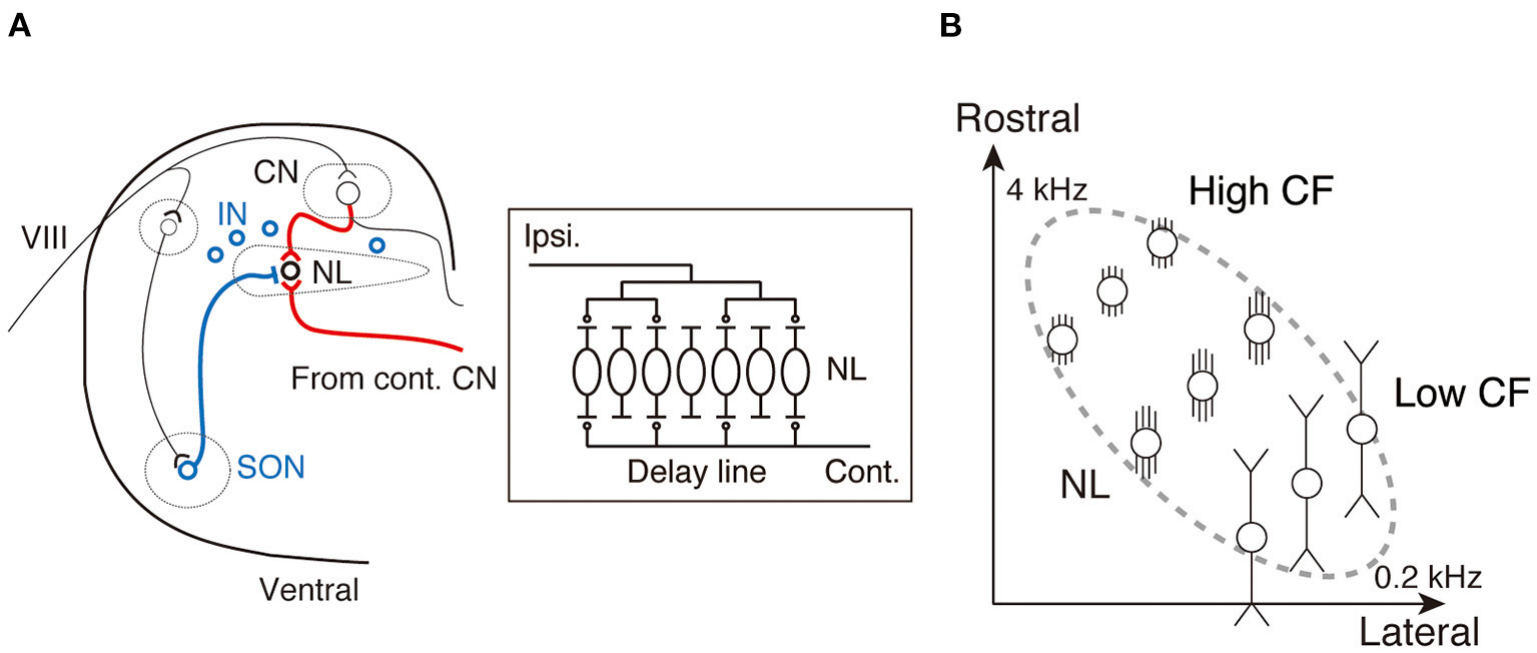
Organization of interaural time difference (ITD) coding circuits in the nucleus laminaris (NL) of the chicken. **(A)** Brainstem auditory circuit of the chicken. Red lines indicate the excitatory projections from the bilateral CN to the NL. Blue symbols and line indicate inhibitory sources (SON and IN) and projection from the SON to the NL, respectively. A schematic drawing of the ITD coding circuit within the NL is shown in the box (right). CN, cochlear nucleus; IN, interneuron; VIII, auditory nerve; SON, superior olivary nucleus. **(B)** Tonotopic organization of the NL.

**Figure 2:**
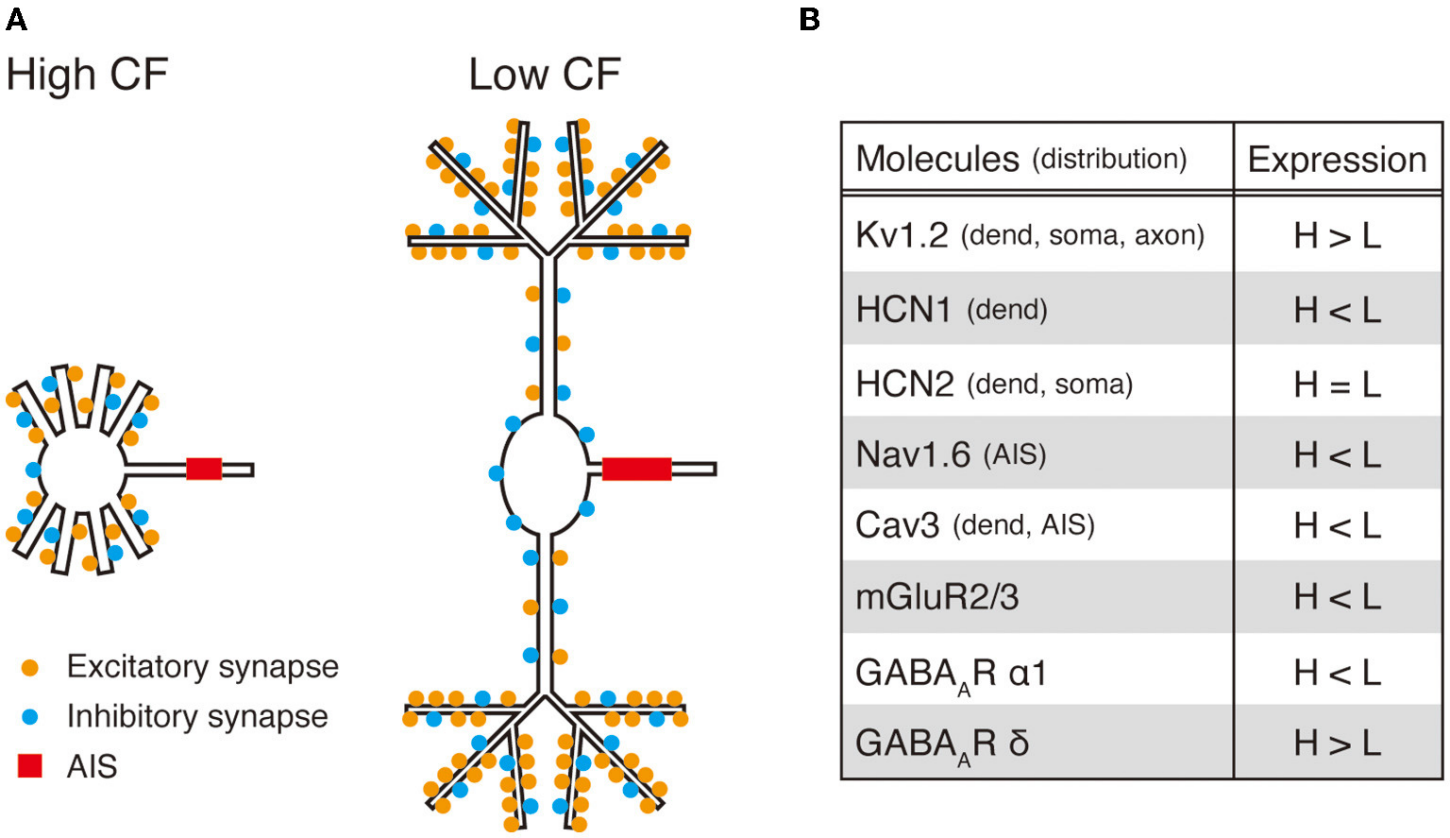
Summary of tonotopic refinements in chick NL neurons. **(A)** Tonotopic differences in excitatory and inhibitory synapses (location and density) and AIS (location and length) are shown in schematic drawings of neurons with high and low characteristic frequency (CF). **(B)** Location of ion channels and receptors and their tonotopic differences in expression are listed.

ITD information is extracted at the 3rd-order brainstem auditory nucleus, the nucleus laminaris (NL) in birds and the medial superior olive (MSO) in mammals, where synaptic inputs from both ears first converge (Klumpp and Eady, 1956; Moiseff and Konishi, 1981; Carr and Konishi, 1990; Overholt et al., 1992; Grothe, 2003). The extraction of ITDs is explained using the classical Jeffress model (Jeffress, 1948), which is based on an array of coincidence detectors innervated by delay lines. Delay lines create systematic delays in the arrival of action potentials within the array, while coincidence detectors generate action potentials when they receive simultaneous synaptic inputs from both ears. In the NL of the chicken, projection fibers from the contralateral cochlear nucleus form delay lines, and NL neurons act as coincidence detectors, which allows the encoding of each ITD as the location of the most active neuron within the nucleus (Figure 1A). The NL is organized tonotopically (Rubel and Parks, 1975), with the CF of neurons increasing from caudo-lateral to rostro-medial direction for the audible frequency range (0.2–4 kHz), and ITDs are processed by frequency-specific neurons (Figure 1B). Accurate coincidence detection in each tonotopic region is a prerequisite for ITD coding (Kuba, 2007). Importantly, NL neurons receive synaptic inputs at a rate corresponding to their CF from each side of the cochlear nucleus during ongoing sound (Funabiki et al., 2011), which is attributed to the convergence of phase-locked inputs from multiple afferents on the neurons, implying that the oscillation frequency of converging synaptic inputs reaches a few kHz for neurons with higher CFs (>1 kHz). Thus, rapid synaptic and membrane responses are expected to be particularly important for neurons with higher CF to integrate binaural signals during ongoing sound. In contrast, for neurons with low CF (<1 kHz), the requirement for speed may be smaller because of the relatively long period of sound waves (Slee et al., 2010); however, it is still critical to maintain integration for wide intensity ranges by adjusting the size of synaptic responses. To meet these requirements and accomplish ITD coding across frequencies, NL neurons are differentiated along the tonotopic axis, which is represented by dendritic morphology, potassium and other active conductance channels, and distributions of excitatory and inhibitory synapses (Figure 2). In the following section, we summarize these differentiations and explain how they optimize frequency-dependent ITD coding in the NL. We will also discuss the similarities and differences between NL and MSO neurons.

## Morphology of Dendrites

NL neurons extend their dendrites in the ventral and dorsal directions and receive excitatory synaptic inputs from each ear on different sides of the dendrites. A model study estimated that this segregation of bilateral inputs is crucial for preventing the generation of action potentials by unilateral inputs (Agmon-Snir et al., 1998). Notably, the length of dendrites differs along the tonotopic axis in the NL; it increases with decreasing CF (Figures 1B, 2A) (Smith and Rubel, 1979; Kuba et al., 2005). Neurons with higher CF have multiple short and thick dendrites, which ensure fast and large EPSPs at the soma by making the entire cell electrically compact and are favorable for the ITD coding of high-frequency sounds. In contrast, neurons with low CF have a smaller number of long dendrites with many distal thin branches, which is compatible with the above model, and their functional role in ITD coding will be discussed in a later section.

In the MSO of the gerbil, dendrites are also long, as in NL neurons with a low CF; however, they are composed of a thick trunk and mostly lack branches (Rautenberg et al., 2009), which reduces dendritic filtering and ensures a fast EPSP time course (Mathews et al., 2010). However, these characteristics might have large variations because MSO neurons with many thin dendritic branches are also observed in gerbils (Bondy et al., 2021) and other mammalian species (Schwartz, 1977; Henkel and Brunso-Bechtold, 1990; Smith, 1995).

## Potassium Conductances

Kv1.2 channels, which mediate the low-voltage-activated dendrotoxin-sensitive K^+^ current, are the major source of conductance activated at rest in NL neurons. These channels are distributed in the soma and dendrites, while they are slightly skewed to the soma (Kuba et al., 2005). A similar distribution of Kv1 channels has been observed in the MSO neurons of the gerbil (Mathews et al., 2010). The expression level of Kv1.2 channels differs among tonotopic regions; it is higher in neurons with higher CF (Figure 2B) (Kuba et al., 2005; Hamlet et al., 2014). Because these channels are strongly activated by EPSPs because of their rapid activation kinetics, they accelerate the falling phase of EPSPs. Thus, the rich expression of Kv1.2 channels together with the faster kinetics of EPSCs accelerates the EPSP time course and ensures the accurate ITD coding in the neurons (Kuba et al., 2005; Slee et al., 2010). In contrast, neurons with low CF show relatively low expression of Kv1.2 channels and slow kinetics of EPSCs, which underlies the relatively slow EPSP time course in the neurons.

Hyperpolarization-activated cyclic nucleotide-gated (HCN) channels, which mediate inward current during membrane hyperpolarization, are preferentially distributed at dendrites and are the other major source of conductance at rest in NL neurons (Biel et al., 2009). Two subtypes, HCN1 and HCN2, are expressed in NL, and their expression levels differ among tonotopic regions; HCN1 expression increases toward lower CFs, whereas HCN2 expression is less graded along the tonotopic axis (Figure 2B) (Yamada et al., 2005). HCN1 channels are activated substantially at rest because of their positive voltage dependence, thereby increasing resting conductance at the dendrites of neurons with low CF. In cortical and hippocampal pyramidal neurons, dendritic HCN channels deactivate during EPSPs and ensure a uniform EPSP time course, irrespective of the input location (Magee, 1998; Williams and Stuart, 2000). In auditory neurons, basal activation of HCN channels has been proposed to accelerate the EPSP time course, although its kinetics is slow (Golding et al., 1995; Yamada et al., 2005). Thus, HCN1 channels in NL neurons with low CF might contribute to adjusting the EPSP time course at dendrites. However, HCN2 channels have more negative voltage dependence and their contribution to the resting conductance is small. Interestingly, noradrenaline causes a depolarizing shift in the voltage dependence of HCN2 channels through the elevation of intracellular cAMP in neurons with high CF (Yamada et al., 2005). This modulation causes membrane depolarization, accelerates the EPSP time course, and improves coincidence detection. Monoaminergic modulation has been reported in the MSO, where serotonin causes a negative shift in the activation voltage of axonal HCN1 channels and increases neuronal excitability (Ko et al., 2016). Therefore, the arousal system of animals may have the potential to regulate the acuity of sound localization with attention.

## Active Conductances

Nav1.6 channels are the major subtype of Na^+^ channels in NL neurons (Kuba et al., 2014). These channels are not distributed at the dendrites and the soma but accumulate at the axon initial segment (AIS) (Kuba et al., 2006), which is the neuronal compartment that mediates the generation of action potentials (Kole and Stuart, 2012). The AIS of NL neurons differs in length and distance from the soma systematically along the tonotopic axis; it is shorter and located more distally toward the high CF (Figure 2A). This differentiation in AIS distribution is crucial for NL neurons to maintain membrane excitability and maximize their response to ITD during ongoing inputs in each tonotopic region (Kuba et al., 2006). In particular, in neurons with high CF, high-rate synaptic inputs lead to plateau depolarization at the soma due to strong temporal summation, which causes inactivation of Na^+^ channels at the AIS and reduces neuronal excitability. However, this effect is relieved by separating the AIS from the soma, which attenuates depolarization at the AIS *via* the filtering effects of the axon. This emphasizes the importance of adjusting the distribution of Na^+^ channels in the AIS for frequency-dependent ITD coding in NL. A tonotopic difference in AIS length has been reported in MSO neurons of the baboon (Kim et al., 2021), but not in the gerbil (Bondy et al., 2021).

T-type Ca^2+^ channels (Cav3 channels) are characterized by their rapid and transient activation around resting potentials (Perez-Reyes, 2003) and mediate augmentation of EPSPs at dendrites in various neurons (Huguenard, 1996; Gillessen and Alzheimer, 1997; Magee and Carruth, 1999; Cain and Snutch, 2010). In the NL, Cav3 channels are distributed at the dendrites of neurons with low CF and augment EPSPs (Figure 2B) (Fukaya et al., 2018). However, this augmentation of EPSPs is limited to the initial phase during repetitive inputs because of the progression of inactivation, suggesting their minimal contributions to dendritic integration and the ITD tuning for ongoing sound. Therefore, Cav3 channels may function as a source of Ca^2+^, which maintains the dendritic structure of neurons through calcium-dependent kinase pathways (Lohmann and Wong, 2005; Redmond and Ghosh, 2005; Nourbakhsh and Yadav, 2021). This might be compatible with the observation that gerbil MSO neurons, which have a rather simple dendritic morphology, lack these channels during the late postnatal period (Franzen et al., 2020). Cav3 channels are also localized in the AIS of NL neurons (Fukaya et al., 2018). The roles of these channels in AIS are unknown, but they may contribute to the reorganization of the AIS structure (Kuba, 2012; Akter et al., 2020).

## Excitatory Synapses

Excitatory synapses are distributed specifically in the dendrites of NL neurons. The pattern of synapse distribution within dendrites critically affects the computation of neurons, and the effects depend on the morphology and biophysical characteristics of the dendrites. For example, in pyramidal neurons, dendrites have strong active conductances, such as Na^+^ channels, Ca^2+^ channels, and NMDA receptors, and clustering of synapses amplifies EPSPs and causes supralinear integration by activating these active conductances (Johnston and Narayanan, 2008; Larkum and Nevian, 2008). In NL neurons, in contrast, this amplification does not occur because these active conductances are weak or absent at dendrites (Kuba et al., 2006; Sanchez et al., 2010; Fukaya et al., 2018). We recently revealed that the distribution of excitatory synapses is coupled with the morphology of dendrites in NL neurons and optimizes ITD coding in each tonotopic region (Figure 2A) (Yamada and Kuba, 2021). In neurons with higher CF, excitatory synapses are distributed uniformly along short and thick dendrites, which minimizes the loss of charge during the integration and propagation of EPSPs along dendrites. Thus, this dendritic synapse geometry in neurons with higher CF maintains the size and shape of EPSPs at the soma, thereby improving ITD coding for high-frequency sound. However, in neurons with low CF, excitatory synapses are clustered in the distal thin part of the long dendrites. This implies that the input site is electrically compact because of the thinness of the dendrites and segregation from the soma. This facilitates spatial summation, causes large depolarization, and increases the loss of charge by reducing the driving force of the synaptic current and increasing the shunting K^+^ current at the input site, which increases the sublinearity of EPSPs at the soma. Since synaptic inputs from each ear are restricted to one side of the dendrites in these neurons, this sublinearity suppresses action potential generation by monaural inputs, while maintaining action potential generation during binaural coincident inputs by preventing saturation of EPSPs, which ensures ITD coding even for strong inputs (Agmon-Snir et al., 1998; Yamada and Kuba, 2021). Notably, this effect does not occur for high-frequency sound (>1 kHz) because extensive temporal summation causes plateau depolarization and suppresses action potential generation, even for binaural coincident inputs. Thus, the long dendrites together with the distal synapse clustering allow fine adjustment of the size of EPSPs in neurons with low CF and work as a mechanism to maintain ITD coding across wide intensity ranges for low-frequency sounds. Activity-dependent regulation during ongoing sound is also prominent in neurons with low CF; activation of metabotropic glutamate receptors (mGluRs) lowers the release probability at presynaptic terminals to reduce synaptic depression (Okuda et al., 2013; Lu et al., 2018) and/or activate the high-threshold Kv channels to follow repetitive firing (Hamlet and Lu, 2016).

In the MSO of the gerbil, excitatory synapses are distributed uniformly along the long dendrites (Couchman et al., 2012; Callan et al., 2021), which would minimize the loss of charge along dendrites and maintain the size and shape of EPSPs at the soma, as in NL neurons with high CF. However, further analyses of the distribution of synapses are needed in other mammalian species, as dendrite morphology differs among species.

## Inhibitory Synapses

GABAergic inhibitory synapses are distributed uniformly in the dendrites and soma in NL neurons. These GABAergic synapses do not cause hyperpolarization and have their effects primarily *via* shunting conductance; they accelerate the time course and decrease the size of EPSPs (Hyson et al., 1995; Funabiki et al., 1998; Yang et al., 1999; Tang et al., 2009). The density of GABAergic synapses differs along the tonotopic axis; it increases toward a low CF (Figure 2A) (Carr et al., 1989; Code et al., 1989; Nishino et al., 2008). In addition, the composition of GABA_A_ receptors also differs; the expression of the α1 subunit is higher toward lower CFs, making the kinetics of IPSCs faster in neurons with low CF (Figure 2B) (Tang and Lu, 2012; Yamada et al., 2013). Consequently, GABAergic inputs have more prominent effects on the ITD coding of NL neurons with low CF (Nishino et al., 2008), while the tonic conductance of extrasynaptic GABA_A_ receptors containing δ subunits is larger in neurons with higher CF (Figure 2B) (Tang et al., 2011). There are two sources of GABAergic inputs to the NL: neurons in the superior olivary nucleus (SON; Lachica et al., 1994; Yang et al., 1999; Burger et al., 2005) and inhibitory interneurons around the NL (Carr et al., 1989; von Bartheld et al., 1989). These GABAergic inputs are driven by auditory nerve activity and provide feedforward inhibition to NL neurons. Thus, they increase with an increase in sound intensity and work as another mechanism for maintaining ITD coding for intensity ranges for low-frequency sounds. Interestingly, however, the pattern of IPSCs differs between the two inputs: inputs from SON neurons cause plateau-like IPSCs (Yang et al., 1999; Coleman et al., 2011), whereas those from interneurons cause phasic IPSCs phase-locked to stimuli (Yamada et al., 2013). Therefore, both GABAergic inputs accelerate and attenuate EPSPs and suppress action potential generation by monaural inputs, while the effects from the SON are substantial for the entire intensity range, and the effects from interneurons are restricted to weak intensity stimuli. This indicates that these two types of inhibitions cooperate with each other and ensure ITD coding from weak to strong intensity inputs for the neurons with low CF. The uniform distribution of these inhibitory synapses within a cell may avoid their accumulation at dendrites, which would otherwise increase the shunting conductance and make EPSPs too small to induce action potentials, even for binaural coincident inputs. Thus, the distributions of excitatory and inhibitory synapses would be particularly important for NL neurons with low CF in adjusting the shape and size of EPSPs and optimizing ITD coding against intensities.

MSO neurons receive feedforward glycinergic inhibitory inputs from both ears at the dendrites and the soma. Because these inhibitory inputs are precisely time-locked to the excitatory inputs, they directly modulate the shape of EPSPs and shift the timing of their peak. These glycinergic IPSPs have been proposed to shift the optimal ITDs for maximal firing (Brand et al., 2002), but their functional roles remain under debate (Joris and Yin, 2007; Roberts et al., 2013; Franken et al., 2015).

## Summary and Conclusion

NL neurons of the chicken show multiple differentiations according to their CF. In neurons with high CF, the rapid EPSP time course is primarily shaped by strong Kv1.2 expression and short dendrites. In addition, the uniform distribution of excitatory synapses, together with fewer inhibitory synapses, prevent extensive loss of charge during the activation of large Kv1.2 conductances. In contrast, in neurons with low CF, the EPSP time course is rather slow, in part because of the weak Kv1.2 expression, as well as long and branched dendrites. However, inhibitory synapses are enriched in the neurons, and excitatory synapses are preferentially clustered at the distal thin part of long dendrites. These characteristics enable the neurons to adjust the size of EPSPs in an intensity-dependent manner, prevent saturation of EPSPs, and ensure the dynamic range of ITD coding, which underlies the intensity tolerance of low-frequency spatial hearing.

MSO neurons of the gerbil encode ITDs of low-frequency sounds and possess long dendrites, like NL neurons with low CF. However, they differ in several ways from NL neurons. First, MSO neurons have large Kv1 currents, lack thin dendritic branches, and distribute excitatory synapses uniformly along dendrites, which accelerates the time course of somatic EPSPs, as observed in NL neurons with high CF. Second, they do not show apparent tonotopic variations in these characteristics (Bondy et al., 2021; but see Baumann et al., 2013). Third, glycinergic inhibitory synapses shape the time course of EPSPs, and their contribution to the size of EPSPs remains elusive. The implications of these differences are unknown, but they may reflect a species difference in the strategy for low-frequency ITD coding and/or in head size between animals. In the mammalian lateral superior olive, which is responsible for binaural spatial hearing of higher frequency sounds, neurons show tonotopic variations in their dendritic morphology and expression of Kv1 channels (Sanes et al., 1992; Barnes-Davies et al., 2004). Therefore, detailed analyses of the lateral superior olive and MSO along the tonotopic axis in other mammalian species may provide a more comprehensive understanding of the sound frequency-dependent computation of ITDs in brainstem auditory circuits.

## Author Contributions

RY and HK wrote the manuscript. Both authors contributed to the article and approved the submitted version.

## Funding

This work was supported by a grant-in-aid from MEXT for Scientific Research (21H02577 to HK) and grants from the Takeda Science Foundation (to RY and HK).

## Conflict of Interest

The authors declare that the research was conducted in the absence of any commercial or financial relationships that could be construed as a potential conflict of interest.

## Publisher's Note

All claims expressed in this article are solely those of the authors and do not necessarily represent those of their affiliated organizations, or those of the publisher, the editors and the reviewers. Any product that may be evaluated in this article, or claim that may be made by its manufacturer, is not guaranteed or endorsed by the publisher.
